# Consequences of Ongoing Civil Conflict in Somalia: Evidence for Public Health Responses

**DOI:** 10.1371/journal.pmed.1000108

**Published:** 2009-08-11

**Authors:** Debarati Guha-Sapir, Ruwan Ratnayake

**Affiliations:** World Health Organization Collaborating Centre for Research on the Epidemiology of Disasters (CRED), School of Public Health, Université catholique de Louvain, Brussels, Belgium

## Abstract

Debarati Guha-Sapir and Ruwan Ratnayake use field data to demonstrate the severe vulnerability faced by much of the Somalian population due to ongoing conflict, and call for concerted public health interventions and access to food aid especially in southern Somalia.

Recurrent civil conflict has blocked progress toward improving health in Somalia. Violent power struggles between political factions followed the breakdown of the government in 1991. Large numbers of civilians were displaced and warlords diverted food aid. In response, a United States-led military intervention attempted to facilitate access for humanitarian relief. While armed forces clashed in Mogadishu, food distribution was disrupted and famine continued in the south. United Nations (UN) peacekeepers followed but were unable to restore order. Of the ensuing period, Alex de Waal wrote that “centralized political authority has never reemerged” [1].

Warfare erupted again in 2006 between the transitional government and insurgents in the southern and central regions, with pockets in Baidoa and Mogadishu under government control [2]. Ethiopian troops entered to quell threats to the border and reinstate the government. UN and African Union peacekeepers also arrived, but were declared to be enemies of the Islamist movement [3]. From 1997 to 2005, Somalia surpassed Sudan, Afghanistan, and Iraq in reports of attacks against aid workers [4]. The UN and many nongovernmental organizations (NGOs) now operate remotely from Nairobi.

The election of a new president and reformulation of parliament in 2009 brings renewed optimism for stabilization. Yet Somalia remains a fragmented region consisting of two effectively independent states (Puntland and Somaliland), a small area governed by transitional authorities and the greater part overseen by Islamist groups.

We believe that the stark lack of humanitarian space and current insecurity has now undermined the impact of humanitarian assistance in Somalia, a country where half of the population is dependent on health relief and food aid. The government has been unable to implement the rule of law, and external aid constantly faces the threat of diversion. Moreover, inter-clan politics ensure that humanitarian engagement remains complex. Sections of the population have been left to deal with the devastating consequences without adequate access to aid.

In this essay, we use field data to demonstrate that geographic segments of the population have chronically faced severe vulnerability and are now facing health threats to their survival. Without the prioritization of basic but crucial public health interventions and negotiated access to food aid, we expect the situation to worsen.

## Navigating a Complex Humanitarian Environment

A little over a million people have been internally displaced since fighting resumed in 2006. The Food Security Analysis Unit of Somalia (FSAU) closely monitors livelihoods, and estimates 3.25 million in need of the most basic emergency food aid [5], a 77% increase since early 2008. The UN's Consolidated Appeal Process is now seeking assistance for nearly half of the population [6]. While the world's media has focused on the rampant piracy in the Gulf of Aden where oil tankers are seized, basic food aid is in danger of diversion every day. During August 2008 when sea escort for the World Food Program was unavailable, only 9,500 of the required 30,000 metric tons of food aid were delivered, causing major delays and impacting over one million persons [7]. Further threats of crop failure, drought, flooding, and escalating food prices are omnipresent.

## Mortality as an Indicator of Humanitarian Need

The absence of central governance means that vital registration systems have ceased to exist. Nevertheless, the scale of the conflict's impact on civilians must be monitored in order to identify where humanitarian needs are most pressing. UNICEF's national survey of 2005 highlights the dangers already faced by children with a demographic mortality rate of 135 deaths per 1,000 children under the age of five years [8]. This is well above rates in neighboring Ethiopia and Kenya. With only 29% of Somali children under two years of age immunized against measles, this is no small wonder.

Death rates have become widely used as an indicator of the impact of violent events on civilian populations and the progress of humanitarian operations. Retrospective mortality surveys, many conducted by the FSAU and NGOs, give insight into the impact across geographic regions of Somalia. The measures used are crude death rates (CDRs), defined as deaths per 10,000 midterm population per day; and under-5 death rates (U5DRs), defined as deaths per 10,000 midterm children under 5 years of age per day. Although mortality surveys conducted by relief agencies during humanitarian crises are not reviewed by institutional review boards, they must adhere to informed consent protocols [9],[10].

To assess mortality trends, we examined CDRs and U5DRs since 2002. Death rates were reported in mortality surveys conducted among displaced persons and residents at different geographic levels: camps for displaced persons, cities, districts, provinces, and livelihood zones of nomadic communities. We have adjusted the dates to reflect the midpoint of the mean recall period for which deaths were recorded (the recall periods for four surveys were not reported and therefore charted by the end dates). We graphed results by time and mapped the most recent results by the geographic level represented by the survey.

These surveys represent the collection of mortality surveys compiled in the Complex Emergency Database (CE-DAT; http://www.cedat.org/) [11]. CE-DAT was initiated in 2003 to compile and standardize data on mortality and nutrition of populations affected by complex emergencies and to improve evidence-based policy on conflict prevention and response. Sample surveys are added to the database after the completeness of the report and the quality of the methodology are assessed.

## Mortality Data across Time and Space

**Figure 1:**
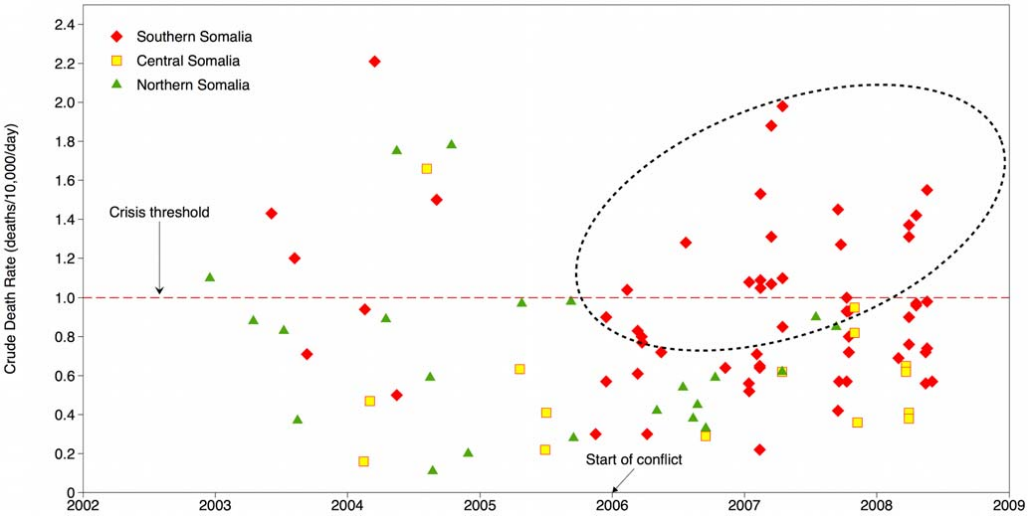
Crude death rates in Somalia, 2002–2008.

For mortality results with respect to time, we charted death rates among three regions (southern, central, and other regions) and noted the eruption of intense fighting in early 2006 (Figures 1 [CDR] and 2 [U5DR]). Comparisons of death rates over time from the conflict-affected south (defined as the region south of Galgadud and identified by the points in red) and comparatively stable center and north raise alarm (yellow and green points respectively). Since the onset of fighting, there has been a surge in mortality rates above the commonly used thresholds for a crisis situation of 1/10,000/day (CDR) and 2/10,000/day (U5DR), reflecting a critical health situation (encircled). In regions less affected by frequent conflict in the center and north, mortality rates have remained poor or acceptable but have come close to reaching emergency thresholds.

**Figure 2:**
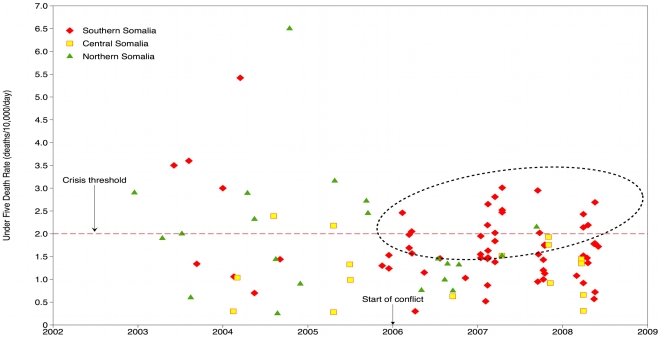
Under-five death rates in Somalia, 2002–2008.

The magnitude of the death rates is high but the fact that the severity has remained elevated since 2006 is unusual for any humanitarian crisis. The high rates coincide with a series of spiraling political events: division within the transitional government, recurrent episodes of heavy fighting, the exit of NGOs, the entry of Ethiopian troops, and military engagement of peacekeepers. These ruinous events have created the “shrinking and deteriorating” operating environment that has confounded relief efforts [7].

**Figure 3:**
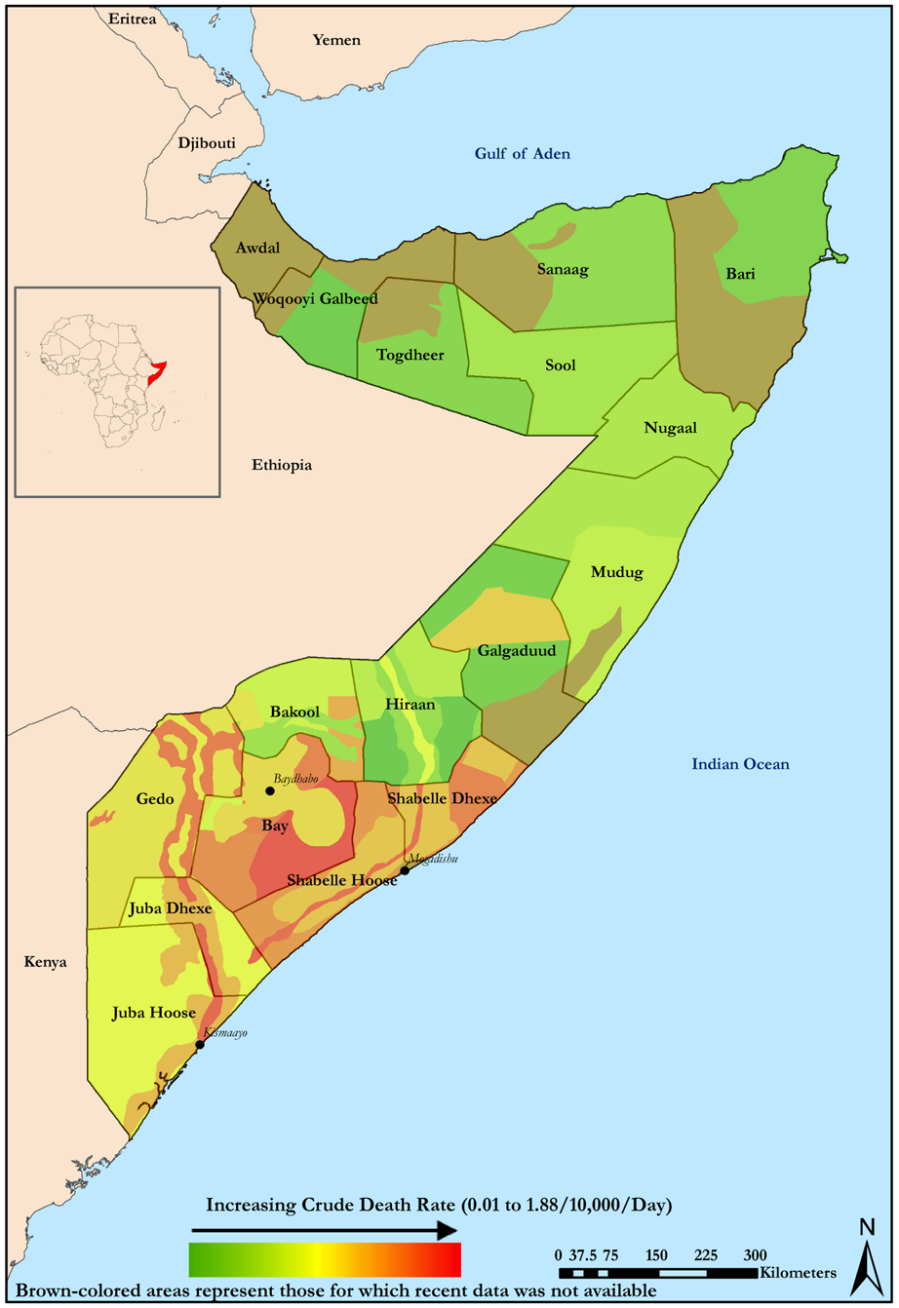
Severity of CDR by area surveyed, latest values in CE-DAT. Credit: David Hargitt.

With respect to space, analyses can illustrate the geographic focus of the critical situation. Mapping of data indicates the severity of the most recent CDR values by areas surveyed (Figure 3). In some areas, CDR has bordered on 2/10,000/day, indicating a situation that is out of control. In the southern and central regions, which are heavily affected by conflict, death rates above threshold have persisted. The highest rates are found primarily in the southern regions of Juba, Shabelle, Bay, and Gedo. Of note, Mogadishu and Kismaayo, located in Lower Juba and the base of the Union of Islamic Courts, are located nearby and are regular sites of clashes.

In Somalia, riverine agriculture and pastoral husbandry strategies are integral to the economy [1]. These agricultural livelihood groups use distinct strategies and assets that direct them across internal borders. During persistent internal conflict, agricultural livelihoods may become unsustainable. The FSAU monitors mortality among these livelihood groups; they are represented on the map by the nonuniform strips that cross provincial borders (Figure 3). Many of these areas clearly represent narrow corridors of stress.

## Interpreting Causes of Death

Survey respondents in the Shabelle and Gedo regions reported that killings or physical injuries were a leading cause of death for CDR estimates above the crisis threshold. Reports noted that this influx of violent deaths coincided with episodes of violence in Mogadishu and internal displacement to these southern regions. However, in other regions and time periods, malnutrition, preventable disease, and birth complications predominated as the main reported causes of child and adult deaths. These are well-known symptoms of poorly functioning health, public health, water, and sanitation systems; mass displacement; and ruptures in the food supply. Most of the population depends on food aid, health systems are localized with little central authority, and there is a vacuum of primary health care workers [12]. A widespread epidemic of clinically diagnosed acute watery diarrhea/cholera across the center and south throughout 2007 and 2008 is further proof of the need to reinforce basic systems [13].

## Focusing on Public Health Priorities

Concise overviews of field survey data can be used as quantitative evidence to identify priorities for the humanitarian response and to monitor the impact of operations. The populations most in need of relief are clearly pooled around specific regions in the south. However, a key determinant of the magnitude of need has been the restrictions on humanitarian access in this region. While aid would be more effective if targeted toward these populations, safe and unhindered humanitarian access has been elusive.

Perhaps most importantly, we note that the main causes of death mirror those of other complex emergencies. Deaths due to malnutrition and preventable disease will continue to predominate over those due to violence. Crumbling public health systems and limited humanitarian relief exacerbate the situation. Vaccine-preventable diseases continue to be a leading cause of death and morbidity among children [13]. The lack of centralized delivery to communities through a working public health system necessitates ad-hoc delivery to accessible populations and results in less than 40% of children having full immunization coverage (DPT3). Intensified programs, such as the WHO/UNICEF's successful Measles Catchup Campaign (2005–2007), conducted deep within southern communities, could greatly prevent child mortality levels from rising further.

It is clear that political realities will continue to either impede or facilitate opportunities for humanitarian intervention. The recent withdrawal of the Ethiopian troops may open the window for genuine intra-Somali dialogue. While political negotiations take place, the need for food and very basic health care continues to grow in the southern and central provinces. The Deyr 2008–2009 season rains were below normal, deepening severe drought and the pastoral food crises in the central regions. Fortunately, another child and mother health campaign launched by WHO/UNICEF in December 2008 endeavors to reach 1.5 million children [14]. In the past, such health interventions have been facilitated by local leaders [15]. Even in such volatile contexts, well-resourced health strategies that are delivered through local partners may still provide effective delivery of preventative health care for women and children at greatest risk. Although nearly 3 million people will continue to need humanitarian aid, our analyses indicate that the success of these programs in the south is of high importance. This reasoning is not only based on the acute needs but it will also to allow people to remain in their villages—a lesson learnt from past similar situations where displacement typically increased the risk of mortality and morbidity.

Somalia presents a complex political picture, and the international community's strategy in the last decade and a half has not yielded encouraging results. The memories of the serious failure of the allied forces and the UN to restore order in 1993–1995 are still rankling in both the regional and international communities. Peacekeepers and the military should not have been sent then to solve a political problem and they should not be sent now. As Ethiopia retreats and a new parliament is formed, the UN should broaden the dialogue to include the leaders of the Islamist groups, who are powerful and there to stay. The same mistakes of excluding the local power brokers in matters of health protection and waiting until children die in food distribution lines outside of Mogadishu should not be made again.

In 1995, one of us argued that the failure of humanitarian intervention during 1992 and 1993 should be a “lesson to the world” [16]. At that time, the international community was ready to provide nearly US$1.5 billion as emergency assistance, in one year. This was well above the amount they were willing to give as development aid for the 10 previous years. Today, the international community is once again ready to spend copiously for policing the international waters off the coast to prevent ransom and the piracy of goods, but are less prepared to spend a tenth of that amount for avoiding severe famine and vaccine-preventable disease among civilians in the south. Indeed, death rates have now reached levels close to those found among populations displaced from Mogadishu during the war in 1992 [17].

Mortality surveys have the potential to highlight the progress of the humanitarian operation, rather than merely to “chronicle an on-going disaster” [18]. Therefore, we have illustrated ways in which this data can provide an evidence base for health priorities in humanitarian assistance to ensure its effectiveness in reducing human and economic costs when resources are shrinking. We would also like to underline the consequences of neglecting the need for social stabilization and long-term development aid in favor of allowing a situation to deteriorate until an emergency occurs or the global economy is affected.

## Abbreviations

CDR: crude death rate
CE-DAT: Complex Emergency Database
FSAU: Food Security Analysis Unit of Somalia
NGO: nongovernmental organization
U5DR: under-5 death rate
UN: United Nations
